# Cardiovascular risk and response to lipid lowering therapy in patients with HIV infection according to different recommendations

**DOI:** 10.1371/journal.pone.0244675

**Published:** 2020-12-29

**Authors:** Agnieszka Pawlos, Marlena Broncel, Ewelina Wlazłowska, Elżbieta Jabłonowska, Paulina Gorzelak-Pabiś

**Affiliations:** 1 Department of Internal Diseases and Clinical Pharmacology, Medical University of Lodz, Lodz, Poland; 2 Department of Infectious Diseases and Hepatology, Medical University of Lodz, Lodz, Poland; University of Tampere, FINLAND

## Abstract

**Background:**

HIV patients are at increased cardiovascular risk while available European cardiovascular recommendations are ambiguous.

**Methods:**

Retrospective analysis of 389 HIV-patients was conducted. Cardiovascular risk was determined by D:A:D, Framingham and SCORE scales. Patients were divided into risk groups as recommended by EACS 2019, PTN AIDS 2019 and ESC/EAS 2019 Guidelines and hypolipemic treatment was evaluated.

**Results:**

In total, 389 HIV-positive patients took part in the study, most of whom were men (n = 312, 80.4%), mean age 41.69±10years. Mean lipid levels among all HIV patients: Tch:177.2±36mg/dl, HDL:48.9±18mg/dl, LDL:103.8±31mg/dl, TG:143.3±81mg/dl, AIP:0.45±0.3, non-HDL:129.2±36 mg/dl. Most of the participants (n = 360, 92.5%) were assigned to the high cardiovascular risk group according to ESC/EAS and PTN AIDS guidelines. The achievement of therapeutic LDLs according to ESC/EAS was 10.3% for those at very high cardiovascular risk (8.7% on lipid lowering treatment vs. 16.7% without hypolipemic drugs) and 12.0% (5.8% treated vs. 13.6% untreated) at high cardiovascular risk; according to PTN AIDS,17.2% achievement was noted by the very high-risk group (13% treated vs. 33.3% untreated), and 45.9% for the high-risk group (37.7% treated vs. 48.0% untreated); according to EACS Guidelines, 2.5% achievement in secondary prevention (3.8% treatedvs. 0% untreated) and 24.7% in primary prevention (22.2% treated vs. 26.1% untreated). Mean doses of statins were 8.75mg±6mg (Rosuvastatin) and 22.35±19mg (Atorvastatin).

**Conclusions:**

The achievement of therapeutic LDLs by all recommendations is unsatisfactory, and generally worse in patients on lipid lowering therapy. Hypolipemic treatment of our HIV patients is based on low doses of statins, even in secondary prevention.

## Introduction

According to a WHO report from the end of 2019, there are 38 million people infected with HIV in the world [1]. In recent years, antiretroviral therapy (HAART), has become highly effective, and extended the lifespan of patients with HIV infection [2]. HIV infection is now seen as a long-term, chronic condition [3]. However, this improved life expectancy has contributed to a tripling of HIV-associated cardiovascular diseases observed over the past 20 years. HIV-associated cardiovascular diseases not only affect mortality, but are also responsible for 2.6mln disability-adjusted life-years (DALYs) per year [4]. Nowadays, among people with HIV infection, the number of AIDS-related and non-AIDS related deaths is nearly equal: 19% of the deaths of HIV-positive people in 2017 due to non-AIDS defining conditions were caused by cardiovascular disease. The mean age at time of death due to cardiovascular disease in this population was just 46 years [5].

It is estimated that people with HIV experience twice the risk of cardiovascular diseases compared to the general population [6], characterized by more advanced and accelerated progression of subclinical atherosclerotic lesions [7]. **However, cardiovascular recommendations for HIV patients provided by the European AIDS Clinical Society (EACS), Polish AIDS Society (PTN AIDS) and European Society of Cardiology/European Atherosclerosis Society (ESC/EAS) differ from each other and thus there are no unequivocal guidelines for this group of patients [8–10].**

The aim of the study was to examine the problem of cardiovascular risk among HIV patients and to highlight the need for this group to take part in cardiovascular disease screening, to monitor lipid parameters and commence lipid lowering therapy where necessary.

The purposes of the study were:

- Cardiovascular risk estimation in HIV patients by D:A:D, SCORE and Framingham scales
- Evaluation of therapeutic goal achievement and lipid lowering therapy as recommended by EACS 2019 Guidelines, PTN AIDS 2019 Recommendations and ESC/EAS 2019 Dyslipidemia Guidelines.

## Materials and methods

The study was conducted between 3^rd^ November 2018 and 20^th^ January 2020 and included 389 patients of the Acquired Immunodeficiency Outpatient Clinic at the Bieganski Hostipal in Lodz, Poland. The inclusion criteria comprised the following: patients with confirmed HIV infection, age ≥18 years old, regularly visiting Outpatient Clinic and undergoing check-ups, if lipid lowering therapy; lasting ≥ one year. The exclusion criteria comprised the following: lack of regular check-ups or lipid lowering therapy for less than a year. The records of the 389 patients were retrospectively analyzed for age, sex, lipid levels (Tch-total cholesterol, LDL-low density lipoprotein, HDL-high density lipoprotein, TG-triglycerides), glycaemia, blood pressure, heart rate, HIV parameters, coexistence of other chronic diseases (cardiovascular diseases, arterial hypertension and diabetes), experience of cardiovascular events (myocardial infarction, stroke, transient ischaemic attack) medications taken for other reasons than HIV, recent antiretroviral treatment, cardiovascular family history, substance use and smoking status. Characteristic of the study group considering lipid lowering therapy can be found in Table 1.

**Table 1:** Baseline characteristic of study group regarding lipid lowering therapy.

	Baseline characteristic of study group regarding lipid lowering therapy		
	HIV patients on lipid lowering therapy n = 92	HIV patients without lipid lowering therapy n = 297	p value
**sex, %male**	n = 81 88%	n = 231 77.8%	**0.0355**
**mean age [years]**	49.21±11	39.27±9	**<0.0001**
**mean BMI**	27.25±5	24.7±4	**0.0001**
**mean LDL [mg/dl]**	108.5±34	102.2±30	0.3147
**The achievement of therapeutic LDLs provided by ESC/EAS 2019 Dyslipidemia Guidelines**	n = 6 6.5%	n = 38 13.6%	0.0926
**The achievement of therapeutic LDLs provided by PTN AIDS 2019 Guidelines**	n = 29 31.5%	n = 133 47.7%	**0.0076**
**The achievement of therapeutic LDLs provided by EACS 2019 Guidelines**	n = 7	n = 12	0.4510
**HIV duration time [years]**	8.98±6	8.96±7	0.4770
**age at HIV diagnosis [years]**	40.37±11	30.3±8	**<0.0001**
**current ARV therapy duration time [months]**	19.58±16	15.68±15	**0.0169**
**ARV treatment duration [months]**	54.88±37	48.48±39	0.2187
**mean SCORE scale [%]**	3.82±3	2.18±2	**<0.0001**
**mean D:A:D [%]**	4.3%±3	1.8%±2	**0.0003**
**mean Framingham [%]**	9.9%±8	8.9%±7	0.6947
**systemic hypertension**	n = 41 44.57%	n = 50 16.95%	**<0.0001**
**diabetes mellitus**	n = 8 8.7%	n = 5 1.7%	**0.0034**
**ischemic heart disease**	n = 9 9.78%	n = 2 0.68%	**<0.0001**
**Current smoking**	n = 49 56.3%	n = 132 48.2%	0.2181
**Current substance use (alcohol, drugs)**	n = 11 12.36%	n = 40 14.55%	0.7260
**Acute Coronary Syndrome**	n = 7 7.6%	n = 0 0%	**<0.0001**
**Ischaemic Stroke**	n = 1 7.6%	n = 7 0.3%	**0.0002**
**TIA**	n = 1 0.1%	n = 0 0%	0.2317

Data is presented as percentage and number of patients or mean ± SD. P value of less than 0.05 is considered significant. Abbreviations: BMI- body mass index; ARV-antiretroviral; TIA–Transient Ischaemic Attack.

### EACS guidelines, ECS/EAS and PTN AIDS recommendations

**Fig 1:**
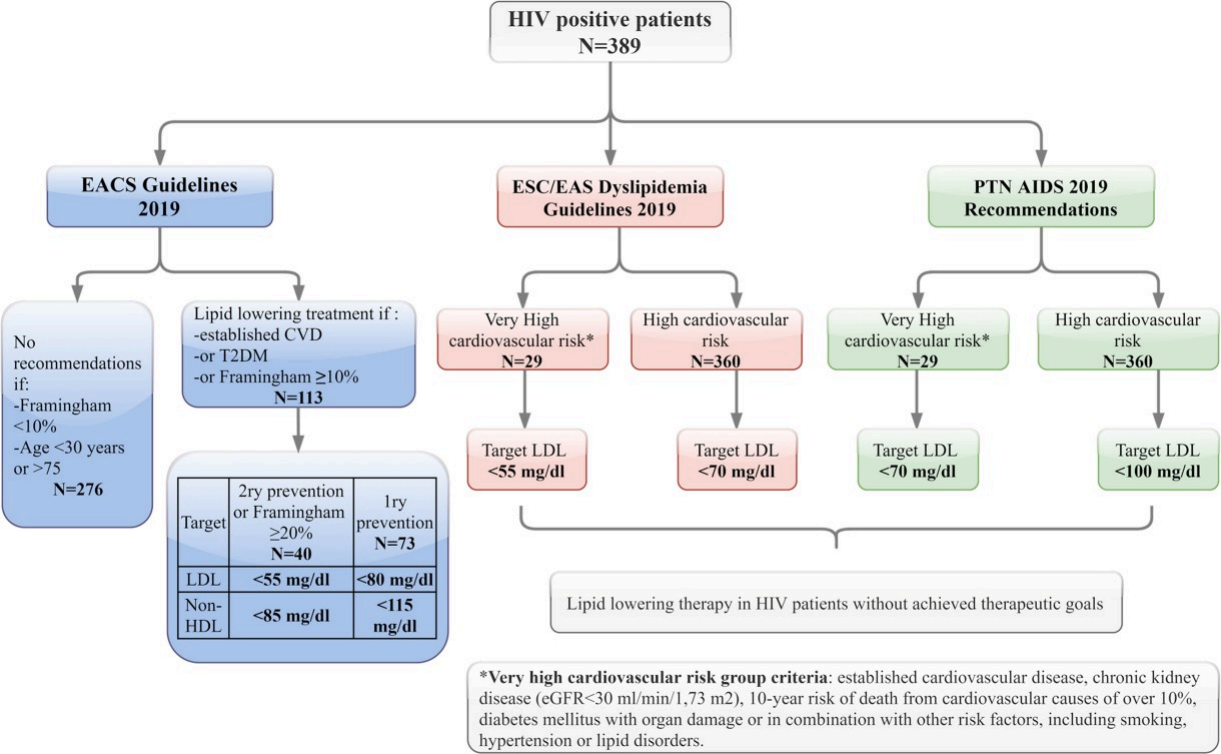
HIV patients according to different cardiovascular recommendations.

All HIV patients included in our study were analyzed as recommended by EACS, ESC/EAS and PTN AIDS. The PTN AIDS guidelines recommend that all HIV patients without other risk factors be considered as high-risk patients. Although the ESC/EAS guidelines do not specify that directly, they advise that lipid lowering therapy should be considered in HIV patients with dyslipidemia to achieve therapeutic LDL defined for high-risk patients; hence, all HIV patients without other risk factors should also be treated as high-risk patients. In the EACS guidelines, no recommendations are given for the following criteria: Framingham <10%, age <30 or >75 years; however, lipid lowering therapy is recommended in cases where established cardiovascular disease or T2DM or Framingham ≥ 10%. More detailed criteria regarding cardiovascular risk groups and therapeutic goals, as stated by different recommendations, are given in Fig 1.

### Cardiovascular risk scales and tools

Cardiovascular risk was estimated using the SCORE (Systematic COronary Risk Evaluation), the D:A:D (the Data Collection on Adverse Effects of Anti-HIV Drugs Study) and the Framingham scale.

**The SCORE scale** was calculated using SCORE Charts for the Polish population [11]. In line with PTN AIDS and ESC/EAS recommendations, all HIV patients without other risk factors were assigned to high cardiovascular risk group.

**The D:A:D scale** was estimated by means of the EACS online calculator: https://www.chip.dk/Tools-Standards/Clinical-risk-scores [12]. The D:A:D (F) full and (R) reduced models were applied as recommended [13].The high cardiovascular risk group was set as ≥5%on the basis of the D:A:D scale.

**The Framingham scale** (evaluating 10 years cardiovascular risk in individuals aged 30–74 years) was calculated following EACS recommendations using official online calculator developed from HIV populations: https://www.chip.dk/Tools-Standards/Clinical-risk-scores [14].

**Non-HDL** was calculated using the following formula:

Non-HDL Cholesterol = Total Cholesterol–HDL cholesterol.

**AIP (Atherogenic index of plasma)** was calculated by means of the formula:

AIP = log (triglycerides/high-density lipoproteins cholesterol) [15–17].

### Statistical analysis

The distribution of the data was verified with the Shapiro-Wilk test. Most variables had other than a normal distribution. The relationships between pairs of groups were tested with Student’s t-test (normal distribution) and the Mann-Whitney U-test (non-normal distribution). All analyses were performed with the STATISTICA v 12.5 software (StatSoft, Inc., Kraków, Poland).

The study was approved by the Bioethical Committee of the Medical University of Lodz, Poland; Consent number: RNN/272/18/KE. Being a retrospective study based on medical records, informed consent from patients was not required. The Bioethical Committee approved this retrospective study without obtaining informed consent from patients.

## Results

A total of 389 HIV-positive patients were included in the study: 312 men (80.4%) and 77 women (19.6%) with mean age 41.69 ±10 years. The mean BMI was 25.27±5 kg. The percentage of patients with normal BMI (18.5–24.99kg/m2) was 43.8%. About 56% of patients had BMI ≥25kg/m^2^, including 11.9% obesity. Mean HIV infection time was 8.96±7 years. The mean length of antiretroviral treatment was 49.81±39 months. Ninety-two patients were receiving lipid-lowering therapy, typically statins such as rosuvastatin or atorvastatin, for longer than one year. Antiretroviral therapy was mostly based on a three-drug regimen consisting of two Nucleoside/nucleotide reverse transcriptase inhibitors (NRTIs) with one of the following: a non-nucleoside reverse transcriptase inhibitor (NNRTI), a protease inhibitor (PI) boosted ritonavir (r)/cobicistat (COBI) or an integrase inhibitor (InSTI). The most common antiretroviral therapy among HIV patients was: Emtricitabine (NRTI) + Tenofovir Alafenamide (NRTI) + Elvitegravir (InSTI) COBI n = 74. Most frequently used antiretroviral drug was Tenofovir Alafenamide (TAF) n = 233.

Eleven participants (2.8%) suffered from ischemic heart disease, 91 (23.5%) hypertension and 13 (3.4%) diabetes mellitus. The number of current smokers was 181/360 (50.3%); 72/254 patients (28.3%) quit smoking. A significant cardiovascular family history was observed in 35 patients (13.5%). Fifty one patients (18.5%) declared current substance use such as drugs, alcohol.

### Cardiovascular risk estimation

#### D:A:D and Framingham scales

**Fig 2:**
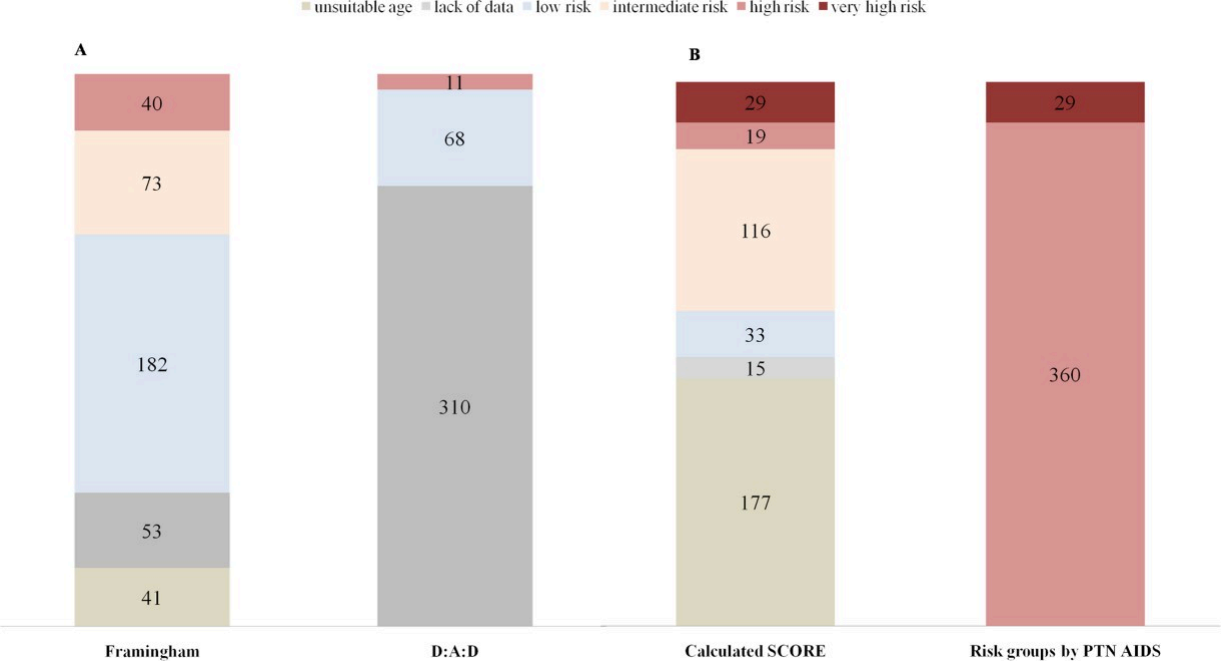
**(A) Percentage distribution of HIV-infected patients in Framingham and D:A:D.** Low, moderate, and high cardiovascular risk according to the D:A:D scale (<5%- low cardiovascular risk; >5%- high cardiovascular risk), Framingham (<10%- low cardiovascular risk, 10%–20%- moderate cardiovascular risk and ≥20%- high cardiovascular risk) **(B) Comparison of distribution of HIV patients between cardiovascular risk groups by calculated SCORE and by PTN AIDS Recommendations.** Low cardiovascular risk SCORE <1%, Intermediate cardiovascular risk SCORE 1–5%, High cardiovascular risk SCORE 5–10%, Very high cardiovascular risk SCORE ≥10%.

Due to insufficient data, the D:A:D scale was applied for only 79/389 (20.9% patients). Mean D:AD was: 2.36%±2. Most patients (86%)demonstrated a D:A:D score below 5%, i.e. not a high cardiovascular risk (Fig 2A). Nearly half of patients had low cardiovascular risk estimated by Framingham.

#### SCORE scale

According to the PTN AIDS guidelines, SCORE scale is not recommended for primary prevention: patients should be qualified to high cardiovascular risk groups unless they meet the criteria of very high cardiovascular risk. Most participants (360/389) were qualified to the high cardiovascular risk group, and the remainders (29/389) to the very high cardiovascular risk group (Fig 2B). As a comparison with the risk group qualification proposed by PTN AIDS analysis, the SCORE scale value was also calculated; however SCORE scale is only validated for patients aged 40–65 years and as the present group is relatively young (mean age 41.69 ±10 years) it could not be applied for 177 (45.5%) of the group (Fig 2B).

### Mean lipid parameters in HIV patients with regards to PTN AIDS and ESC/EAS guidelines and lipid lowering therapy

**Fig 3:**
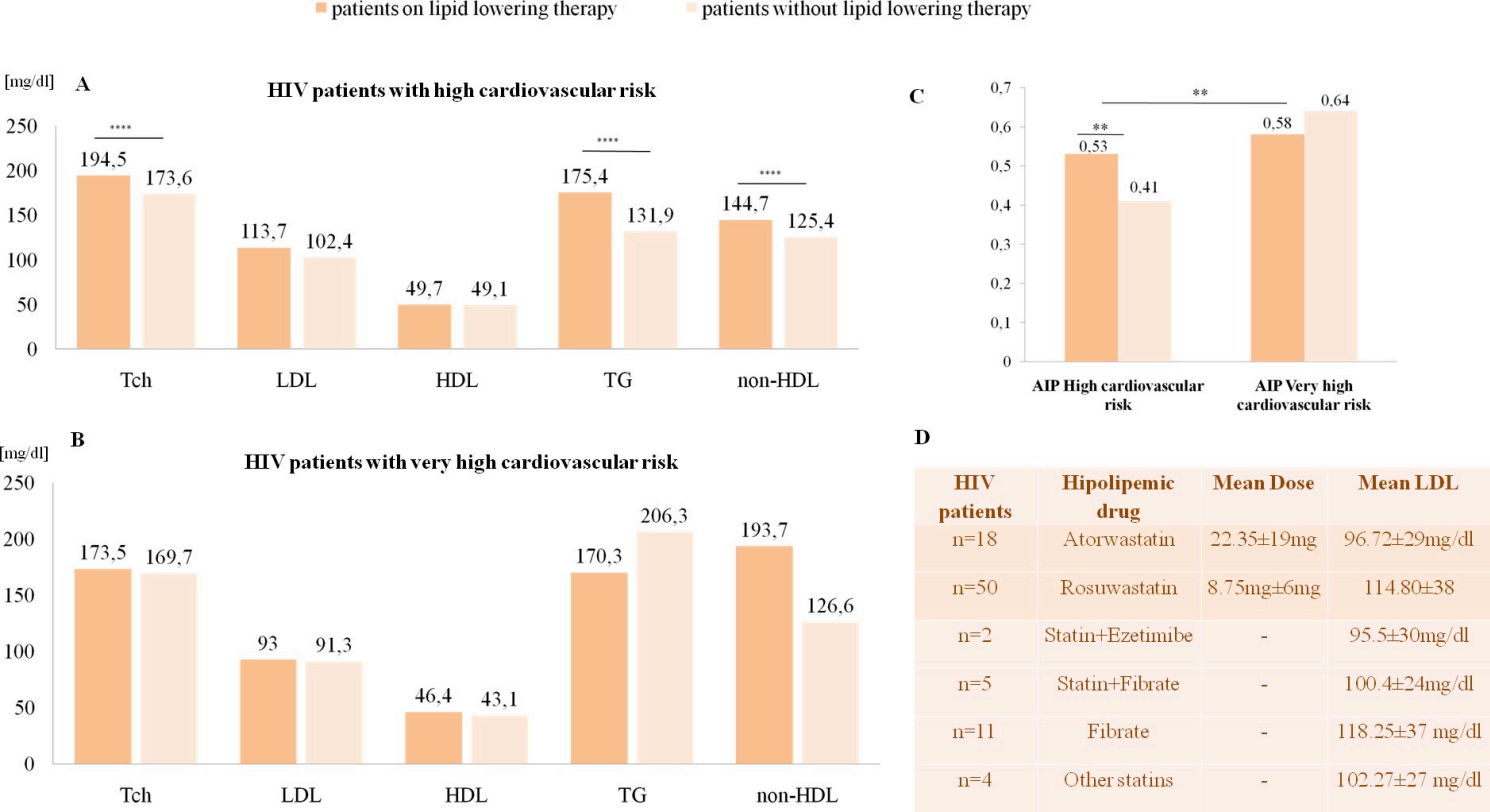
**(A) Mean lipid parameters in patients with high cardiovascular risk**. All patients in high cardiovascular risk (n = 360), those on lipid lowering therapy (n = 69) and those without lipid lowering treatment (n = 297). Tch–Total Cholesterol, TG–Triglycerides. The exact p-values were: Tch**p<0.0001,** LDL p = 0.0584, HDL p = 0.9164, non-HDL **p<0.0001,** TG **p<0.0001(B) Mean lipid parameters in patients with very high cardiovascular risk.** All patients at very high cardiovascular risk (n = 29) on lipid lowering treatment (n = 23) and without lipid lowering therapy (n = 6). Tch–Total Cholesterol, TG–Triglycerides. Exact p-values were: Tch p = 0.8236, LDL p = 0.8870, HDL p = 0.7567, non-HDL p = 0.9800, TG p = 0.7437**(C) Mean Atherogenic Index of plasma, differences between high and very high cardiovascular risk groups with regard to lipid lowering treatment.** The exact p-values were AIP p = 0.0122 for high CV risk patients vs. very high CV risk patients; AIP p = 0.7050 for very high CV risk patients with lipid lowering therapy vs. those without; AIP p = 0.0038 for high CV risk patients with lipid lowering therapy vs. those without. All values are given in [mg/dl] Statistical significance is marked as ** and ****. **(D)** Lipid lowering treatment type along with mean LDL values.

The following mean lipid parameters were calculated among all HIV patients included in the study: Tch 177.2 ±36mg/dl—95% CI (173.5;180.9), HDL: 50.28±31mg/dl—95% CI (47.06; 53.5), LDL 103.8±31mg/dl- 95% CI (100.6; 106.9), TG 143.3±81mg/dl—95% CI (135.1; 151.4), AIP 0.45±0.3–95% CI (0.42; 0.48), non-HDL 129.2±36mg/dl—95% CI (125.5; 133.2). On the basis of PTN AIDS and ESC/EAS guidelines, the participants were divided into two groups based on cardiovascular risk; high risk—360 patients (92.5%) and very high risk—29 patients (7.5%). Of the 29 patients qualified as very high risk CV patients, 14 were associated with a cardiovascular incident, five with T2DM+additional risk factor, four with SCORE≥10%, four with ischaemic heart disease and two with established cardiovascular disease. Both groups were then divided into two subgroups based on their use of lipid lowering therapy. An analysis of the mean lipid values in the two cardiovascular risk groups with regard to lipid lowering therapy is given in Fig 3A and 3B. Interestingly, among HIV patients with high cardiovascular risk, individuals on lipid lowering treatment had significantly higher total cholesterol, triglyceride and non-HDL levels than patients with no lipid lowering treatment (Fig 3A).

#### Atherogenic index of plasma

As HIV patients often experience disturbances in TG and HDL levels, only LDL estimation seems insufficient; therefore, the study included an analysis of the atherogenic index of plasma. HIV patients with very high cardiovascular risk (defined by PTN AIDS and ESC/EAS) were also found to have significantly higher AIP levels than those at high cardiovascular risk. Among the patients with a high cardiovascular risk, those on lipid lowering therapy had significantly higher AIP than those with no hypolipemic treatment (Fig 3C).

#### Lipid lowering therapy

Indications to switch to lipid lowering therapy in HIV patients differ between the analyzed recommendations (Fig 1). According to EACS, 113 of our patients have indications to statins, and only 53 (46.9%) are on lipid-lowering drugs. Regarding other available Recommendations it is respectively 32.7%—PTN AIDS high cardiovascular risk; 79.3%—PTN AIDS very high cardiovascular risk; 22.6%—ESC/EAS high cardiovascular risk and 79.3%—ESC/EAS very high cardiovascular risk.

Generally, out of 389 patients, 92 were currently on lipid lowering therapy. Only 29 (31.5%) of them reached therapeutic LDLs from PTN AIDS 2019 Guidelines (<100mg/dl and <70mg/dl). Barely 6/92 patients (6.5%) managed to achieve therapeutic LDLs from ESC/EAS 2019 Guidelines. Further details regarding lipid lowering therapy and mean LDL values are given in Fig 3D. None of the patients were on PCSK9 inhibitors.

### Achievement of therapeutic goals advised by EACS, ESC/EAS and PTN AIDS recommendations

#### EACS guidelines 2019

**Fig 4:**
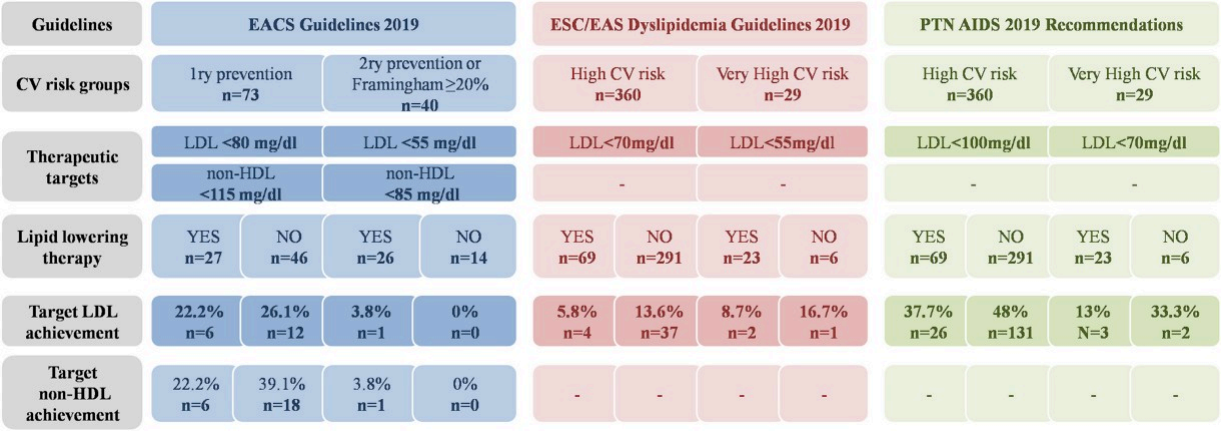
The achievement of therapeutic goals stated in EACS Guidelines 2019, ESC/EAS Dyslipidemia Guidelines 2019 and PTN AIDS Guidelines 2019 with regard to lipid lowering therapy. In the high cardiovascular risk group without lipid lowering therapy 18 patients did not have LDL monitored.

The achievement of therapeutic goals given by EACS Guidelines 2019 regarding lipid lowering therapy is given in Fig 4. Only 113 of the 389 (29%) patients were found to be in line with the guidelines. EACS provides no recommendations for our HIV patients who aged <30 or >75 and/or have Framingham score <10% (n = 276/389; 70.9%). The overall achievement of therapeutic LDL values according to EACS Guidelines was found to be 16.8% [95% CI (9.9%; 23.7%)]—(2.5% in secondary prevention and 24.7% in primary prevention). For the patients who achieved target LDL, the mean LDL value was 61.44±13mg/dl 95% CI (55.14; 67.75) for primary prevention; only one patient achieved target LDL for secondary prevention.

#### ESC/EAS dyslipidemia guidelines 2019

The achievement of therapeutic LDLs advised by ESC/EAS in HIV patients with high and very high cardiovascular risk regarding lipid lowering therapy is given in Fig 4. ESC/EAS Guidelines can be applied to all HIV patients, regardless of age. The overall achievement of therapeutic LDLs was found to be 11.9% [95% CI (8.6%; 15.1%)]—(10.3% in the very high cardiovascular risk group and 12.0% in the high cardiovascular risk group). Among patients who achieved target LDL, the mean LDL values were 49±4mg/dl 95% CI (38.17; 59.83) for those with very high CV risk and 58.12±9mg/dl 95% CI (53.37; 58.88) for high cardiovascular risk.

#### PTN AIDS guidelines 2019

PTN AIDS recommendations could also be applied for all HIV patients. A detailed presentation of therapeutic goal achievement in the high and very high cardiovascular risk groups with regard to lipid lowering therapy is given in Fig 4. The overall achievement of PTN AIDS therapeutic LDL targets was found to be 43.7% [95% CI (38.6%; 48.7%)] - 17.2% in the very high cardiovascular risk group and 45.9% in the high risk group. For patients who achieved target LDL, the mean LDL scores were 55±9mg/dl 95% CI (44.10; 65.90) for those with very high CV risk and 78.61±16mg/dl 95% CI (76.12; 81.09) for those with high cardiovascular risk.

### Cardiovascular incidents

Of the 389 studied HIV positive patients, 14 (3.6%) had experienced 17 cardiovascular incidents (eight ischemic strokes, eight Acute Coronary Syndromes, one TIA). Mean age during cardiovascular incident was 41.8±6 years. The current mean LDL in those patients was 93.14±21 mg/dl and only one of them had achieved therapeutic LDL <55mg/dl. Regarding hypolipemic treatment, 6/14 patients were taking Atorvastatin (mean dose 30 mg) and 6/14 patients were on Rosuvastatin (mean dose 17.5mg). Two patients were also receiving 10mg of ezetimibe, and another two fibrate combined with the statin. One patient was receiving 20mgsimvastatin. One HIV patient after ischemic stroke with LDL 80mg/dl did not receive any lipid lowering treatment. The mean HIV duration time in those patients at the moment of cardiovascular incident was 10.3±6 years. Four patients had experienced a cardiovascular incident before HIV recognition and three patients had cardiovascular incidents and HIV diagnosis in one year.

## Discussion

This study highlights inconsistencies between cardiovascular guidelines for HIV patients given by EACS, ESC/EAS and PTN AIDS. It describes the current status of cardiovascular risk among HIV patients attending the Acquired Immunodeficiency Outpatient Clinic in Łódź, as well as their achievement of therapeutic LDLs given by different recommendations and evaluates lipid reduction. Recent knowledge about cardiovascular disease management and prevention among HIV patients is based only on observational studies. The first-ever randomized controlled clinical trial (REPRIEVE) aimed at the prevention of cardiovascular diseases among HIV patients is currently ongoing [18].

### The challenge of cardiovascular risk assessment in the HIV population

Chronic inflammation, opportunistic infections and the use of some antiretroviral drugs, as well as lifestyle, cigarette smoking, dominant male sex and high alcohol consumption significantly increase cardiovascular risk among HIV infected patients [19]. It is important to note that half the HIV patients in our study report active smoking, which is twice that of the general Polish population (24%) [20]. HIV infection has not been included in commonly used cardiovascular scales (Framingham, SCORE). This is an important point as chronic inflammatory processes exacerbate atherogenesis in HIV positive patients. In addition, the HIV population is relatively young (41.69±10 years in our patients), which significantly limits the possibility of SCORE scale usage (validated for age 40–65 years). In our study, there were 174/389 (44.7%) patients aged under 40 years.

PTN AIDS facilitates the use of SCORE scale, based on which it recommends the assignment of all HIV patients without other risk factors to the high cardiovascular risk group, i.e. 92.5% of patients in our study, with the other 7.5% being assigned to the very high cardiovascular risk group. The lower age limit in the Framingham scale (40–74 years) allows broader screening of the HIV population, but still it does not consider HIV infection; however, only the Framingham risk equation developed from the HIV population could be applied. The Framingham calculations indicated that nearly half the HIV patients in our study (46.8%) had low cardiovascular risk. However, studies on large cohorts of HIV infected men showed that this commonly-used scale underestimated cardiovascular risk among PLWH [21].

The only scale validated for cardiovascular risk assessment in HIV carriers is the D:A:D scale. Despite being suitable for HIV positive patients aged 18–75 years, it is not practical and is difficult to use. To calculate the five-year risk of developing cardiovascular disease, cumulative NRTIs (Nucleoside/nucleotide reverse transcriptase inhibitors) and PIs (Protease inhibitors) exposure is required. This requirement unfortunately makes the D:A:D scale almost useless in everyday outpatient clinical practice, because tools used in screening should be easy to apply. Due to insufficient data, the D:A:D could only be applied only to 20.3% of the studied HIV patients. The findings indicate that only 2.83% of HIV patients were in the high cardiovascular risk group. The mean D:A:D score in our population was 2.37%: markedly higher than the only other available study, which identified a mean score of 0.6% among HIV adults in Africa. This lack of mean D:A:D values among HIV patients demonstrates its impracticality.

### Lipid parameters among HIV patients

Dyslipidemia is a common comorbidity among PLWH [22]. In our study we assessed mean lipid parameters in HIV patients with regard to cardiovascular risk category and lipid lowering therapy. Our results show that in both cardiovascular risk groups, HIV patients on hypolipemic treatment have generally worse lipid parameters than those not receiving treatment. However, the difference is statistically significant only in patients with high cardiovascular risk for total cholesterol, triglycerides and non-HDL. This may prove that current lipid lowering therapy among HIV patients is insufficient.

### Analysis of achievement of therapeutic LDLs

The achievement of target LDLs, regardless of recommendations, among our HIV-positive patients remains unsatisfactory, which is in keeping with van Zoest et al [23]. The highest overall achievement of therapeutic LDL was observed with regard to PTN AIDS 2019 recommendations (43.7%), followed by the EACS guidelines (16.8%) and then the ESC/EAS guidelines (11.9%). According to all given recommendations, HIV patients in very high-risk groups or in secondary prevention find it harder to reach therapeutic targets than high-risk patients. Interestingly, regarding the high cardiovascular risk groups and primary prevention, there is a noticeable tendency that HIV patients who take lipid lowering drugs have worse therapeutic LDL achievement than those without lipid lowering therapy. This might be a consequence of lack of patient adherence, unwillingness to adopt lifestyle changes and the presence of unhealthy eating habits (45% of patients had BMI above the normal limit). It is worth adding that half of our patients (50.3%) were current smokers. In addition, the fact that lipid lowering therapy in patients on antiretroviral treatment is complicated by drug interactions could also have an influence. It has been currently reported, that switch from Tenofovir Disoproxil (TDF) to Tenofovir Alafenamide (TAF) worsens lipid parameters among HIV patients [24,25]. In our study, TAF was the most commonly used antiretroviral drug. Finally, such poor achievement may be due to inadequate lipid lowering treatment, which may be caused either by poor adherence or relatively late onset of statins in insufficient doses. According to our results, limited number of patients eligible for lipid lowering therapy were actually treated. The most common mistake in lipid lowering therapy based on statins is so called ‘therapeutic nihilism’ which is mainly caused by insufficient doses of statins [26]. Bednasz et al also reports low rate of lipid-lowering drugs use among HIV patients [27]. Among our patients, mean statin doses were found to be 8.75±6mg of Rosuvastatin (max. dose 40mg) and 22.35±19mg of Atorvastatin (max. dose 80mg). However, although all given recommendations warn about possible interactions between several antiretroviral drugs and statins, they nevertheless advise their use to reach therapeutic targets. In addition, only two patients were receiving ezetimibe combined with a statin.PTN AIDS allows this combination,as well as the use of ezetimibe monotherapy, but interactions with antiretroviral treatment have not been evaluated yet. None of the patients were on PCSK9 inhibitors., which are becoming more frequently used among HIV patients. According to Boccara et al, PCSK9 inhibitor—Evolocumab was well tolerated by HIV patients. The reachability of therapeutic LDL <70 mg/dl in HIV patients on Evolocumab was 73.3% in contrast to 7.9% in placebo group [28].

Using statins brings undeniable cardiovascular benefits for primary and secondary prevention of atherosclerotic cardiovascular disease (ASCVD) events in the general population [29]. According to the IMPACT model, a 39% reduction of cardiovascular mortality among Polish population was achieved by inter alia a mean reduction in cholesterol levels [30]. No such clinical trials have been performed on HIV patients. In particular, the use of statins in young HIV patients without additional cardiovascular risk factors other than HIV arouses controversy. However, lipid lowering therapy among HIV patients has been recommended to contract the effect of hyperlipidemia on the deterioration of neuropsychological parameters [31].

The high efficiency of antiretroviral treatment has prolonged life expectancy and thus the ageing of the HIV patient population, and as such, an increase in age related comorbidities is unavoidable. It is estimated that by 2040, 70% of HIV patients will be >50 years [32]. Currently, among our patients only 19% is older than 50 years and even now they fail to reach therapeutic recommendations. If appropriate steps are not taken now, the number of cardiovascular diseases will certainly grow among HIV patients.

### Cardiovascular incidents

Despite being a relatively young population (41.69±10 years in our study) HIV positive patients experience cardiovascular events. Croxford et al. report that mean age during death due to cardiovascular incidents in HIV patients was 46 years old. The mean age for experiencing non-fatal cardiovascular incidents among our HIV patients was 41.8±6 years.

To achieve therapeutic LDL reduction, ESC/EAS recommend using statins, at the maximal allowed doses of 40mg of Rosuvastatin or 80mg of Atorvastatin [10]. In addition, the pragmatic approach to atherosclerotic cardiovascular disease risk assessment and prevention of HIV infection stated by the AHA advises initiation of statin therapy in low doses among HIV patients, even in secondary prevention [33]. Only two out of our fourteen HIV patients who had experienced cardiovascular incidentshad statins in maximal doses. Interestingly one patient after cardiovascular incident had no lipid lowering treatment. Our results correspond with those of Boccara et al, who noticed that HIV patients are “resistant to statin” and are less likely to achieve therapeutic goals after cardiovascular incidents than HIV-negative individuals [34]. Similar difficulties have also been reported by Burkholder GA et al, who found that less than 10% of HIV patients on lipid lowering therapy had ≥ 50% reduction of LDL level [35].

## Conclusions

- Available cardiovascular recommendations for HIV patients given by EACS, ESC/EAS and PTN AIDS are ambiguous with regard to risk group classification, therapeutic goals and criteria for the inclusion of lipid-lowering therapy.
- Therapeutic LDLs given by each set of recommendations were achieved by a low percentage of patients: EACS– 16.8%, PTN AIDS– 43.7%, ESC/EAS– 11.9% overall achievement. This finds may be related to the use of statins at low doses and rarely combining statin with ezetimibe.
- For the high CV risk HIV patients on lipid-lowering therapy, the use of the 70mg/dl LDL target provided by ESC/EAS 2019 guidelines and the 100mg/dl LDL target provided by PTN AIDS recommendations increases the achievement proportion from 5.8% to 37.7%. This difference highlights the need to standardise the CV Guidelines for HIV patients.
- In the studied group, lipid lowering therapy was based on low doses of statins, even in secondary prevention; this is inconsistent with ESC/EAS Guidelines, but consistent with the AHA Scientific Statement.
- There is no unified, practical and easy to use cardiovascular risk assessment tool appropriate for use in HIV patients:
  ○ The D:A:D scale requires 13 variables and could not be used in 79.7% of our HIV patients due to insufficient data.
  ○ The Framingham scale could not be used in 24.2% of HIV patients due to lack of data and unsuitable age.
  ○ Calculated SCORE scale could not be applied to 45.5% HIV patients due to unsuitable age.
- Comparing the guidelines for prevention and treatment was problematic: the ESC and PTN treatment guidelines have different cut-off values in LDL outcomes, resulting in different targeted LDL achievement (%). This value might not necessarily be associated with CV outcomes, such as myocardial infarction and stroke.

### Limitations

In the end, some limitations of the study should be noted. First, we did not analyze antiretroviral treatment in detail. Secondly, our data is based only on medical records, we have no information about exact adherence to lipid lowering treatment. Unfortunately, due to lacking data, we were able to calculate the D:A:D scale in a limited number of patients.

## Supporting information

S1 Data (XLSX)
