# Correction: Cardiovascular risk and response to lipid lowering therapy in patients with HIV infection according to different recommendations

**DOI:** 10.1371/journal.pone.0246176

**Published:** 2021-01-22

**Authors:** The PLOS ONE Staff

## Notice of republication

An incorrect version of Supporting information file S1 Data was published in error. The publisher apologizes for this error. This article was republished on January 13, 2020 to correct for this error. Please download this article again to view the correct version.
